# Phenotypic Heterogeneity of Patients With Marfan Syndrome in Puerto Rico: A Case Series

**DOI:** 10.7759/cureus.68791

**Published:** 2024-09-06

**Authors:** Gabriel A Jiménez-Berríos, Sebastián J Vázquez-Folch, Natalio Izquierdo

**Affiliations:** 1 Department of Ophthalmology, School of Medicine, Universidad Central del Caribe, Bayamón, PRI; 2 Department of Surgery, School of Medicine, Medical Sciences Campus, University of Puerto Rico, San Juan, PRI

**Keywords:** autosomal dominant, ectopia lentis, fbn1 gene, fibrillin, marfan syndrome

## Abstract

Purpose: Previous studies have reported on the cardiovascular, ocular, and musculoskeletal findings in patients with Marfan syndrome (MFS). This study aims to report the ocular and genotypic findings in patients with the syndrome in Puerto Rico.

Patients and methods: A chart review of a cohort of patients with the syndrome from Puerto Rico was done. Patients were examined by at least one of the authors (NJI). Fibrillin-1 (*FBN1*) full gene sequencing was done to all patients (Laboratory for Molecular Medicine, Center for Genetics and Genomics, Cambridge, MA). This study was approved by the Institutional Review Board of the Universidad Central del Caribe (approval number: 2024-07).

Results: Six patients aged 28-79 years were examined. There were seven female and three male patients. The average visual acuity was 0.49 and 0.52 in the right eye (OD) and left eye (OS), respectively. The average refraction (spherical equivalent) was -1.28 sph OD and -1.07 sph OS. The average intraocular pressure was 14 mmHg in both eyes (OU). A patient had a dislocated lens OD; a patient had lens dislocation OU; and a patient had prosthesis OD and aphakia OS. Upon optical coherence tomography (OCT), the retinal nerve fiber layer (RNFL) average was 75.86 µm OD and 81.85 ​​µm OS; the average cup-to-disc (C/D) ratio was 0.41 and 0.35 in the right and left eye, respectively. Upon visual field testing, the average mean deviation (MD) was -6.27 dB OD and -8.55 dB OS.

Conclusions: Our findings underscore the significant phenotypic and genotypic heterogeneity of patients with MFS in Puerto Rico. The identification of several mutations in the *FBN1* gene in the Puerto Rican population demonstrates the need for an up-to-date approach to diagnose and co-manage patients with the syndrome. This study contributes to a deeper understanding of the genetic heritage of patients with the syndrome and highlights the potential for personalized therapeutic interventions.

## Introduction

Patients with Marfan syndrome (MFS) have a genetic disorder that primarily affects connective tissues. Its prevalence is about one in 5,000 individuals [1]. It is inherited as an autosomal dominant trait [1]. Patients with the syndrome have cardiovascular, ocular, and musculoskeletal manifestations. Clinical diagnosis relies on the revised Ghent criteria [2], emphasizing the importance of early detection and co-management to mitigate life-threatening cardiovascular risks, such as aortic aneurysms and dissections [1]. 

Ocular findings in patients with the syndrome have been reported extensively [3]. Findings in patients with the syndrome include elongated axial length (myopia), strabismus, cornea plana, glaucoma, ectopia lentis, and retinal detachment [4,5]. 

Musculoskeletal manifestations in patients with the syndrome include long extremities, arachnodactyly, dolichostenomelia, positive wrist and ulnar signs, scoliosis, pectus excavatum or carinatum, and pes planus [6].

Dietz and co-workers were the first to identify the* *fibrillin-1 (*FBN1*) gene mutations associated with the syndrome [7]. Significant expression of *FBN1* mRNA is observed in various tissues such as the cultured fibroblasts, subcutaneous adipose tissue, aorta and coronary arteries, esophagus, tibial nerve, and ovaries [8]. The amount of *FBN1* in the human eye varies per person [9]. It is mostly found in ciliary zonules which if mutated can lead to ectopia lentis [3].

Alterations in *FBN1* play a role in the development of numerous diseases in humans. Interestingly, both a decrease and an increase in *FBN1* levels can enhance the availability and signaling of transforming growth factor-beta (TGF-β) [8]. In pathological circumstances, imbalanced *FBN1* can either stimulate tumor cell growth or trigger apoptosis in endothelial cells [10].

There is a paradoxical effect, where diminished *FBN1* levels due to variants trigger TGF-β pathway activation, contributing to the advancement of fibrillinopathies [11]. To support this theory, experiments involving the administration of TGF-β antagonists have been shown to exert a protective, anti-apoptotic effect in the lungs of mice with *FBN1* deficits [12]. This indicates that *FBN1*'s control over TGF-β signaling might fluctuate depending on genetic factors or the presence of disease, highlighting the intricate and context-specific nature of *FBN1*'s actions [8].

We report on the ocular and genotype findings of six patients with MFS in Puerto Rico. 

## Materials and methods

A chart review of a cohort of patients with the syndrome from a single center in Puerto Rico was done. Patients were selected based on a set of inclusion criteria. All subjects were diagnosed with MFS according to the revised Ghent criteria, which include a combination of clinical manifestations, family history, and genetic testing. Patients were examined by at least one of the authors (NJI). *FBN1* full gene sequencing was performed for all patients (Laboratory for Molecular Medicine, Center for Genetics and Genomics, Cambridge, MA). Patients of all ages were included if they had a positive *FBN1* gene mutation test result. These six patients underwent a comprehensive ophthalmic examination. The examination included intraocular pressure (IOP) measurement, optic nerve coherence tomography, and visual field testing, among others. We focused specifically on individuals residing in Puerto Rico to understand the regional manifestations and variations of MFS. This study was approved by the Institutional Review Board of the Universidad Central del Caribe (approval number: 2024-07). 

## Results

Six patients aged 28-79 years were examined, of which four were females and two were males as summarized in Table 1.

**Table 1:** Genetic mutations and ophthalmic examination findings of the six patients FBN1: fibrillin-1; OD: right eye; OS: left eye; IOP: intraocular pressure; SPH EQ: spherical equivalents; PR: Puerto Rico

Age	Genetic mutation	City	Gender	Visual acuity	Refraction	SPH EQ	Retinal fiber layer	Average cup-to-disk ratio	Visual field testing mean deviation	IOP
40	FBN1 (c.4532G>A p.Cys1511Tyr)	Canovanas, PR	Male	OD 0.2; OS 0.3	OD -2.75 +1.00×95˚; OS -2.00 +0.25×180˚	OD -2.25; OS -1.87	OD 83 µm; OS 81 µm	OD 0.07; OS 0.08	OD -6.79 dB (p<0.5%); OS -7.41 dB (p<0.5%)	OD 15; OS 15
79	FBN1 (c.1633C>T p.Arg545Cys)	San Juan, PR	Female	OD 0.5; OS 0.4	OD +1.50 +0.75×100˚; OS +3.25 +1.00×100˚	OD +1.88; OS +3.75	OD 93 µm; OS 43 µm	OD 0.48; OS 0.14	OD -21.78 dB (p<0.5%); OS -17.96 dB (p<0.5%)	OD 13; OS 14
71	FBN1 (c.1633C>T p.Arg545Cys)	San Juan, PR	Female	OD prosthetic; OS 1.3	OD-OS -1.00 +0.50×180˚	OD-OS -0.75	OD-OS 89 µm	OD-OS 0.30	OD-OS -28.63 dB (p<0.5%)	OD-OS 13
37	FBN1 (c.4532G>A p.Cys1511Tyr)	Canovanas, PR	Female	OD 0.6; OS 0.3	OD -9.00 +2.50×20˚; OS -9.50 +2.00×160˚	OD -7.75; OS -8.50	OD 86 µm; OS 80 µm	OD 0.45; OS 0.36	OD -9.92 (p<0.5%); OS -12.03 (p<0.5%)	OD 12; OS 12
28	FBN1 (c.4001G>A p.Gly1334Asp)	San Juan, PR	Female	OD 0.0; OS 0.1	OD -2.50 +1.00×105˚; OS -3.00 +1.25×75˚	OD -2.00; OS -2.375	OD 95 µm; OS 96 µm	OD 0.60; OS 0.58	OD -1.92 dB (p<10%); OS -1.80 dB (p<10%)	OD 14; OS 12
47	FBN1 (c.8054A>G p.His2685Arg)	San Lorenzo, PR	Male	OD 0.4; OS 0.2	OD -11.50 +5.50×120˚; OS -7.00 +4.50×70˚	OD -8.75; OS -4.75	OD 73 µm; OS 83 µm	OD 0.62; OS 0.63	OD -8.96 dB (p<0.5%); OS -2.65 dB (p<0.5%)	OD 15; OS 15

The best corrected visual acuity (LogMAR) ranged from 0.0 to 0.6 in the right eye (OD) (avg=0.34) and 0.1 to 1.3 in the left eye (OS) (avg=0.43). Refraction (spherical equivalent) ranged from -8.75 to +1.875 (avg=-3.78) and -8.50 to +3.75 (avg=-2.41) in the right and left eye, respectively. IOP had a range of 12-15 OD (avg=13.8) and 12-15 OS (avg=13.5). Three out of the six patients presented with bilateral lens subluxation. One patient presents with a prosthetic right eye. The retinal fiber layer ranged from 73 µm to 93 µm (avg=86 µm) and 43 µm to 96 µm (avg=78.7 µm) in the right and left eye, respectively. The average cup-to-disc (C/D) ratio ranged from 0.07 to 0.62 (avg=0.44) and 0.08 to 0.63 (avg=0.35) in the right and left eye, respectively. The visual field testing mean deviation (MD) ranged from -21.78 to -1.92 OD (avg=-9.86) and -28.63 to -1.80 OS (avg=-11.75). 

## Discussion

Gehle and co-workers reported that patients with MFS had worse visual acuity when compared to their control group. Gehle et al.'s patients had an average visual acuity of 0.13 logMAR. Our patients' visual acuity was an average of 0.34 logMAR OD and 0.43 logMAR OS. This finding is slightly different than previously reported visual acuity in patients with the syndrome. This difference highlights the importance of regular ophthalmic checkup and optimal refraction in patients with the syndrome [13].

In Gehle et al.'s study, 67.4% of patients with MFS had a myopia of >0.75 D, while 31.4% of patients had >3 D. In our series, patients had an average refraction of -3.78 D and -2.41 D in the right and left eye, respectively. These findings are compatible with previous studies. Patients with the syndrome may have axial and lenticular myopia [14]. 

Gehle et al.'s study reported a lower average IOP in patients with MFS compared to the control group [13]. The average IOP of their patients was 14.6 mmHg compared to 15.1 mmHg in the control patients. Our patients had an average IOP of 13.6 mmHg. Patients in our sample have lower average IOP. However, there is a need for regular IOP measurements and glaucoma screening in patients with the syndrome.

Maumenee reported that 60% of patients with MFS have ectopia lentis [15]. In our series, 50% of patients with the syndrome had lens dislocation. These findings are compatible with previous literature. It is important to realize that not all patients with the syndrome have ectopia lentis [16].

Xu and co-workers reported the distribution of macular and optic nerve topography in individuals aged 8-56 years with MFS through the use of spectral domain optical coherence tomography (SD-OCT) [17]. The results showed that 18% of the eyes of patients with the syndrome had a retinal nerve fiber layer (RNFL) thickness in <5% of the population [17]. We report on a cohort of patients between the ages of 28 and 79 who demonstrated an average RNFL thickness of 82 μm. These findings are concordant with Xu and co-worker's cohort study.

Studies done at Northwestern University in Chicago [17] reported that patients with MFS have an average C/D ratio of 0.46. Patients in our sample had a C/D ratio of 0.39. Our findings are compatible. 

Previous studies have reported that patients with MFS have an increased propensity for glaucoma. In our study, upon Humphrey's visual field testing, MD ranged from -21.78 dB to -1.92 dB OD (avg=-9.86 dB) and -28.63 dB to -1.80 dB OS (avg=-11.75 dB). To our knowledge, this is the first report of visual field testing of patients with MFS.

Limitations of the study include a small sample size and a single center. 

## Conclusions

Our findings underscore the significant phenotypic and genotypic heterogeneity of patients with MFS in the Puerto Rican population. The identification of several mutations in the *FBN1* gene in the Puerto Rican population demonstrates the need for an up-to-date approach to diagnose and co-manage patients with the syndrome. This study contributes to a deeper understanding of the genetic heritage of patients with the syndrome and highlights the potential for personalized therapeutic interventions.
